# *miR-451* Deficiency Is Associated with Altered Endometrial Fibrinogen Alpha Chain Expression and Reduced Endometriotic Implant Establishment in an Experimental Mouse Model

**DOI:** 10.1371/journal.pone.0100336

**Published:** 2014-06-17

**Authors:** Warren B. Nothnick, Amanda Graham, Joshua Holbert, Mitchell J. Weiss

**Affiliations:** 1 University of Kansas Medical Center, Department of Molecular & Integrative Physiology, Kansas City, Kansas, United States of America; 2 The Children's Hospital of Philadelphia, Division of Hematology, Philadelphia, Pennsylvania, United States of America; Baylor College of Medicine, United States of America

## Abstract

Endometriosis is defined as the growth of endometrial glandular and stromal components in ectopic locations and affects as many as 10% of all women of reproductive age. Despite its high prevalence, the pathogenesis of endometriosis remains poorly understood. MicroRNAs, small non-coding RNAs that post-transcriptionally regulate gene expression, are mis-expressed in endometriosis but a functional role in the disease pathogenesis remains uncertain. To examine the role of microRNA-451 (*miR-451*) in the initial development of endometriosis, we utilized a novel mouse model in which eutopic endometrial fragments used to induce endometriosis were deficient for *miR-451*. After induction of the disease, we evaluated the impact of this deficiency on implant development and survival. Loss of *miR-451* expression resulted in a lower number of ectopic lesions established *in vivo*. Analysis of differential protein profiles between *miR-451* deficient and wild-type endometrial fragments revealed that fibrinogen alpha polypeptide isoform 2 precursor was approximately 2-fold higher in the *miR-451* null donor endometrial tissue and this elevated expression of the protein was associated with altered expression of the parent fibrinogen alpha chain mRNA and protein. As this polypeptide contains RGD amino acid “cell adhesion” motifs which could impact early establishment of lesion development, we examined and confirmed using a cyclic RGD peptide antagonist, that endometrial cell adhesion and endometriosis establishment could be respectively inhibited both *in vitro* and *in vivo*. Collectively, these results suggest that the reduced *miR-451* eutopic endometrial expression does not enhance initial establishment of these fragments when displaced into the peritoneal cavity, that loss of eutopic endometrial *miR-451* expression is associated with altered expression of fibrinogen alpha chain mRNA and protein, and that RGD cyclic peptide antagonists inhibit establishment of endometriosis development in an experimental mouse model suggesting that this approach may prove useful in the prevention of endometriosis establishment and survival.

## Introduction

Ectopic growth of endometrial stromal and glandular tissue outside of the uterine cavity (endometriosis) occurs in approximately 10% of all reproductive age women, frequently causing pain, dysmenorrhea and infertility [1], [2]. The disease is thought to develop via retrograde release of viable endometrial tissue into the peritoneal cavity during menstruation. However, this process occurs in most unaffected women [3], [4], and it is therefore postulated that additional factors contribute to the pathogenesis of endometriosis. Considerable attention has focused on alterations in eutopic endometrial tissue that may predispose it to extrauterine implantation, survival and proliferation. Recently, microRNAs (miRNAs) have been implicated in the pathogenesis of endometriosis.

miRNAs are a class of small non-coding regulatory RNAs (18–25 nucleotides) that regulate gene expression post-transcriptionally [5], [6] and are proposed to be involved in diverse developmental and pathological processes. miRNAs have been implicated to play a vital role in development, differentiation, cell proliferation and apoptosis [7]. Not surprising, mis-expression of miRNAs have been detected in disease states including cardiovascular pathologies, neural disorders and various cancers [8]. miRNA profiles have been established for endometriosis in both the disease tissue and eutopic endometrium from these as well as control patients without endometriosis [9]–[12]. These investigators have demonstrated that specific miRNAs are both up- and down-regulated in endometriotic tissue as well as in the eutopic endometrium of women with the disease. In the initial report by Pan and colleagues [9], *miR-451* expression was shown to be one of the most significantly reduced (by approximately 50%) miRNAs in eutopic endometrium from women with endometriosis as well as in endometriotic implants from these women compared to women free of the disease. However, a more recent study by Hawkins and colleagues suggested that *miR-451* may be up-regulated in endometriotic tissue [12] but lower in eutopic endometrium (compared to ectopic).

*miR-451* functions in a variety of physiologic events such as cell proliferation [13]–[16], cell differentiation [17]–[19], and cell migration/invasion [13]–[16]. These events are all conducive to the establishment, progression and survival of endometriotic implants within the peritoneal cavity. *miR-451* has been validated to regulate post-transcriptional expression of proteins which, when over-expressed, may modulate these physiological events conducive to endometriotic implant establishment and/or survival such as macrophage migration inhibitory factor (MIF) [13] and 14-3-3 protein zeta (YWHAZ) [20]. Based upon this information, coupled with the finding that *miR-451* expression is reduced in eutopic endometrium from women with the disease [9], we postulated that this reduced expression in eutopic endometrium may enhance the ability of the endometrial tissue to establish ectopically. Alternatively, the reduced *miR-451* expression in eutopic endometrium may be a result of the disease and not contribute to the establishment of the ectopic lesions. To answer these questions and determine if eutopic endometrial *miR-451* plays a functional role in the establishment of ectopic endometrial lesions/endometriosis, we developed a mouse model for endometriosis which utilized mice which were deficient for *miR-451* expression.

## Materials and Methods

### Ethics Statement

All animal procedures for these experiments were approved by the University of Kansas Medical Center Institutional Animal and Use Committee (protocol ACUP #2011-1986) and follow guidelines as suggested in the “Guide for the care and use of laboratory animals” by the National Research Council of the National Academies. All mice were housed within environmentally controlled conditions under the supervision of a licensed veterinarian.

### Mouse model of endometriosis

**Figure 1:**
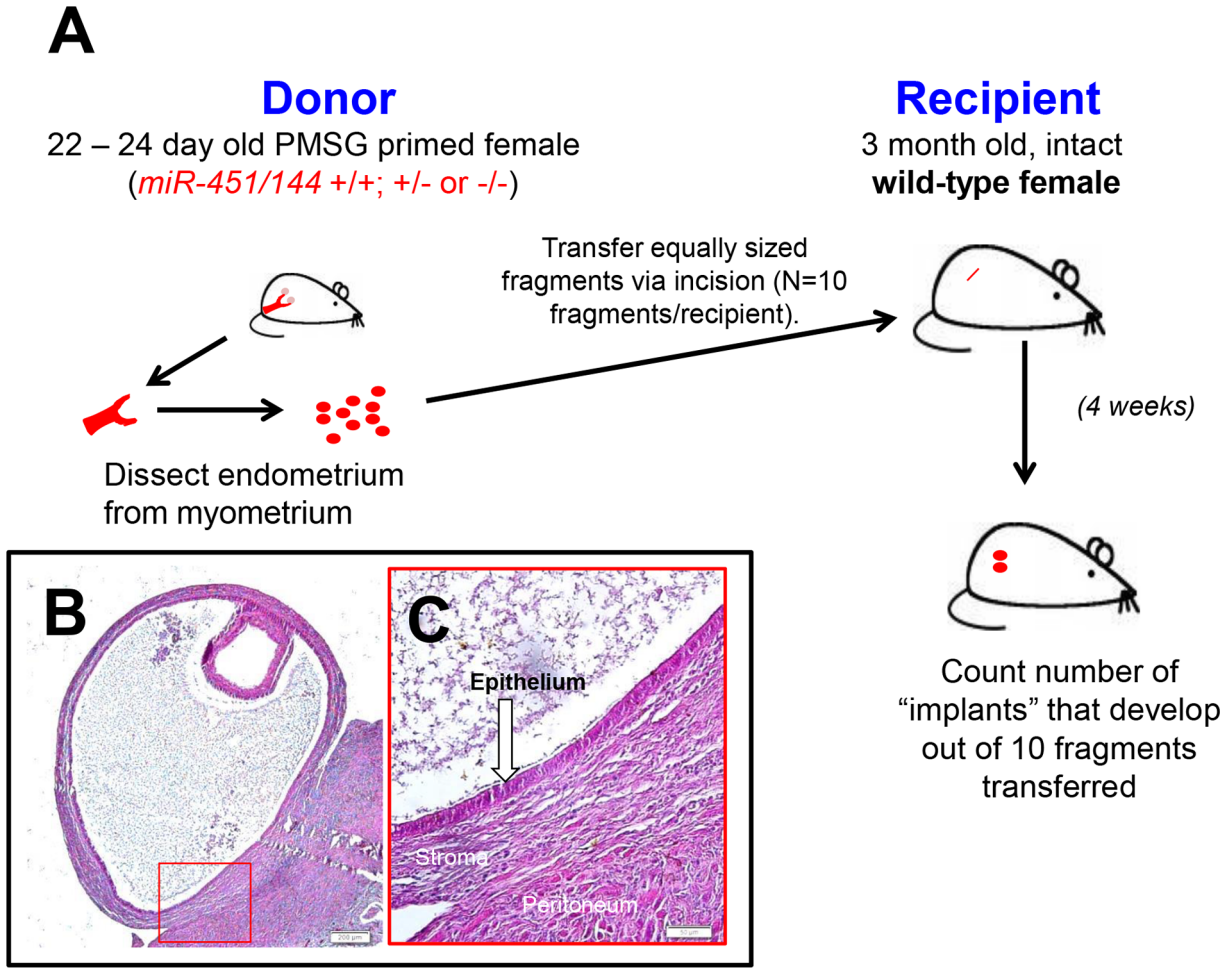
Diagrammatic representation of experimental endometriosis induction in mice and histological representation of ectopic endometriotic implant. A) Diagram depicts donor mice (and genotype) from which endometrial tissue was dissected and separated into myometrium and endometrium (stromal and epithelial components), transfer of 10 equal size (1 mm^3^) endometrial fragments to recipient mice and subsequent assessment of establishment of ectopic “endometriotic implants.” B) histological representation of “endometriotic implant” with red boxed area enlarged in C) white arrow indicates epithelial component and stromal compartment and underlying peritoneum are labeled. Notice the stromal and peritoneal contact at the site of implant attachment. White scale bar  = 50 um.

Endometriosis was experimentally induced using the general approach previously described [21]. Mice were maintained on a 14 L:10 D photo period and provided water and mice chow *ad libitum*. To induce endometriosis, 22–24 day old C57BL/6 female mice were injected s.c. with pregnant mare serum gonadotropin (PMSG; 2 IU; Sigma Chemical Company, St. Louis, MO) to stimulate endogenous estrogen production and subsequent estrogenic response within the uterus. Uteri were then harvested from these donors 42–44 h after PMSG injection. Uterine stroma and epithelium (endometrium) was separated from myometrium with the aid of a dissecting microscope. Endometrial tissue (which contained stromal as well as glandular and luminal epithelium) was cut into 10 fragments of equal size (1 mm^3^). Uterine fragments were suspended in 0.4 mL of sterile saline. Recipient mice (2 to 4 month old wild-type C57BL/6 immuno-competent, reproductively intact females) were anesthetized with ketamine/xylazine and an antibiotic ointment was placed over the corneas to avoid corneal abrasions. The area over the right rib cage was prepared for surgery and a small incision (approximately 0.5 cm) was made exposing the peritoneal cavity. Tissue fragments were injected into the peritoneal cavity through the incision (as depicted in Fig. 1A) and the incision was then closed with wound clips. Carprofen analgesic was given post-operatively at the conclusion of the surgery and again 24 h later. Mice were then sacrificed at indicated time post endometriosis induction. Fig. 1B and 1C represent typical lesion appearance and association of the stromal and peritoneum in implant attachment.

### Endometriosis induction using *miR-451*/*miR-144* deficient mice

*miR-451/144* deficient (*miR-451/144* ^−/−^) and heterozygous (*miR-451/144* ^+/−^) mice on a pure C57BL/6 background were generated as described [22]. A breeding colony was established by crossing *miR-451/144* ^−/−^ male mice with wild-type C57BL/6 females (*miR-451/144* ^+/+^). *miR-451* null or heterozygous females and males exhibit no reproductive abnormalities and exhibit normal fertility. Heterozygous matings then ensued to generate offspring of all three genotypes. Endometriosis was induced by injecting calibrated amounts estrogen-primed endometrial tissue into the peritoneal cavity (as described above). Disease establishment and progression was quantified at various time-points by determining the number of mice with peritoneal implants and the average number of implants/mouse. This was accomplished by sacrificing the animals, opening the peritoneal cavity and then viewing the peritoneal cavity under a dissecting microscope. In this animal model, implants develop on peritoneal surfaces and are easily viewed. Two investigators evaluated each animal for the number of implants per animal and the size of each implant in a blinded fashion.

To follow-up on the initial study, a separate experiment was conducted in which experimental endometriosis was induced as described above with the exception that 2–4 month old wild-type (non-EGFP) hosts (N = 5) received an equal number of wild-type (which expressed EGFP; transgenic mice (C57BL/6-Tg(ACTB-EGFP)1Osb/J; Jackson Laboratories; Bar Harbor, ME) and *miR-451* deficient implants (which did not express EGFP; 8 endometrial fragments per genotype per recipient). The number of implants that developed in each mouse was then counted and genetic background verified by examination of EGFP (wild-type) expression.

### Analytical Two-dimensional DiGE gels

Uterine fragments (stromal and epithelial tissue [devoid of myometrium] identical to that used for endometriosis induction) were isolated from 22–24 day old PMSG-primed wild-type and *miR-451* ^−/−^ females (N = 6/genotype) and frozen on dry ice. Samples were shipped to Applied Biomics (Hayward, CA) for two-dimensional differential in-gel electrophoresis (DiGE) and protein identification by mass spectrometry to establish protein profiles between genotypes and subsequent protein identification. Image scans were carried out immediately following the SDS-PAGE using Typhoon TRIO (GE Healthcare). Scanned images were then analyzed by Image QuantTL software (GE-Healthcare), and then subjected to in-gel analysis and cross-gel analysis using DeCyder software version 6.5 (GE-Healthcare). The ratio change of the protein differential expression was obtained from in-gel DeCyder software analysis. Those proteins whose expression was >1.5-fold in the *miR-451* ^−/−^ uterine fragments compared to wild-type counterparts were then subjected to isolation using Ettan Spot Picker (GE Healthcare) and subsequent MALDI-TOF (MS) and TOF/TOF (tandem MS/MS) using a 5800 mass spectrometer (AB Sciex).

For protein identification, the resulting peptide mass and the associated fragmentation spectra were submitted to GPS Explorer version 3.5 equipped with MASCOT search engine (Matrix Science) to search the database of National Center for Biotechnology Information non-redundant (NCBInr). Searches were performed without constraining protein molecular weight or isoelectric point, with variable carbamidomethylation of cysteine and oxidation of methionine residues, and with one missed cleavage allowed in the search parameters. Candidates with either protein score C.I.% or Ion C.I.% greater than 95 were considered significant. All proteins reported in this work had a C.I.% of 100%.

### Western analysis

Western analysis was performed as previously described [21]. Briefly, total protein was extracted from frozen uteri using RIPA buffer (Cell Signaling Technology, Danvers, MA). Protein concentration in each sample was determined using the *DC* Protein Assay (Bio-Rad Laboratories, Richmond, CA). The same amount of protein (100 µg) was subjected to 4–15% polyacrylamide gel electrophoresis and electroblotted onto nitrocellulose membranes (GE Healthcare, Piscataway, NJ). Rabbit anti fibrinogen alpha chain (Fga; LifeSpan BioSciences, Inc. LS-C159646 1∶500 in 5% BSA at 4C overnight) and sheep anti-mouse secondary antibody (1∶5000, GE Healthcare) were used for Western analysis. Stripping and reprobing for β-actin (Santa Cruz) was conducted to normalize protein expression levels. Immunodetection was carried out using an enhanced chemiluminescence (ECL) kit (Amersham Biosciences, Piscataway, NJ). Fga protein expression was normalized to that of β-actin and the value was expressed as a fold-change from wild-type tissue.

### *miRNA* assessment by qRT-PCR

Quantitative RT-PCR (qRT-PCR) was performed as previously described [23], [24]. Briefly, total RNA was isolated using TRI-Reagent (Sigma Aldrich Chemical Company, St. Louis, MO) according to recommendations of the manufacturer. Total RNA (250 ng in 5 µl) was reverse transcribed using reverse transcription (RT) kits (Applied Biosystems; Foster City, CA) following the manufacturer's protocol with the following modifications. Briefly, miRNAs were reverse transcribed in a single reaction using 2 µl of each miRNA specific 5X RT primers. Resulting material was then used for independent qRT-PCR for *miR-451* and *miR-144* as well as *fibrinogen alpha chain* (*Fga*). RT-PCR was carried out on an Applied Biosystems HT7900 Sequence Detector. To account for differences in starting material, *U6* was used for *miR-451* and *miR-144* while *18S* was used for *Fga*. All primers were purchased from Applied Biosystems. Each cDNA sample was run in triplicate and the relative abundance of each target divided by the relative abundance of *U6* for miRNAs and *18S* for *Fga* in order to normalize for the starting quantity of cDNA. Each primer set included a minus template cDNA control. The delta-delta CT method was used to calculate the fold-change values among samples.

### Plasmin activity assays

Plasmin activity was assessed as previously described [24] with minor modifications using the plasmin-specific chromogenic substrate D-Val-Leu-Lys-p nitroanilide dihydochloride (VLKpNA; Sigma; V7127). Endometrial samples (50 µl volume) were incubated with 950 µl of assay buffer (50 mM Tris buffer (pH 7.4) containing 110 mM NaCl) containing 0.6 mM VLKpNA substrate. Samples were incubated at 37 C for 24 h (time was determined empirically for plasmin activity as was in the linear range). At the end of the incubation period, plasmin activity was determined by reading the samples at 405 nm absorbance. Optical density (OD) was determined and plasmin activity is reported as the change in OD from substrate blanks normalized per mg of protein in each sample.

### Endometrial stromal cell plating assays

The transformed human endometrial stromal cell line (t-HESC; obtained from the ATTC, Manasses, VA, and described in [25]) was cultured in phenol red-free DMEM:F12 supplemented with 10% charcoal-stripped FBS, 1 mM sodium pyruvate and penicillin and streptomycin to near confluence. T-HESC cells were then passed and 1×10^5^ cells/mL were pre-incubated with increasing concentrations of cyclic RGDFV (cyloRGDfV; an RGD containing peptide antagonist [0, 5, 20 or 100 uM; SCP0111; Sigma-Aldrich, St. Louis, MO) in DMEM:F12 media lacking FBS and phenol red. Incubation was carried out at room temperature on a rotating platform. Cells (1×10^5^ cells/ml) were then transferred to 12-well tissue culture plates coated with either vitronectin (VN; 10 µg/mL), fibronectin (FN; 10 µg/mL) or BSA (10 µg/mL) and incubated for 24 h. Cell viability was assessed for all treatments in separate aliquots (not plated) prior to addition to tissue culture plates. Cell attachment, spreading and survival (referred here in as plating) were then checked at 24 h after addition of the cells to each well. To do so, media was removed and the number of non-attached cells was counted using a hemacytometer. To assess the number of cells that remained attached, cells were trypsinized and again counted. The percent of cells plating was then calculated for each treatment using the formula: % plating  =  (number of cells attached)/(number of cells attached + number of non-attached cells) ×100%.

### *In vivo* modulation of endometriosis establishment

To determine if the RGD motif within the fibrinogen alpha polypeptide isoform 2 precursor protein could affect *in vivo* establishment of endometriosis, endometriosis was induced as described under “Mouse model of endometriosis” with the exception that endometrial fragments were placed into sterile PBS containing vehicle (sterile water; N = 8) or cyloRGDfV (100 uM; N = 8). Samples were incubated for 1 h at room temperature with constant rotation, then transferred as described under “Mouse model of endometriosis” and implant establishment was assessed 2 weeks post-induction.

### Statistical analysis

All data were analyzed using GraphPad Instat3 software. Data were analyzed using one-way ANOVA followed by post-hoc analysis using Tukey's LSD method when appropriate. When data did not display a normal distribution, non-parametric analysis was performed (see Figure legends for specific details where appropriate). Significance of association between endometriosis development and treatment group was analyzed using Fisher's exact test. For all assessments, a p-value <0.05 was considered statistically significant.

## Results

### *miR-451* deficiency impairs the establishment of experimentally-induced endometriosis

**Figure 2:**
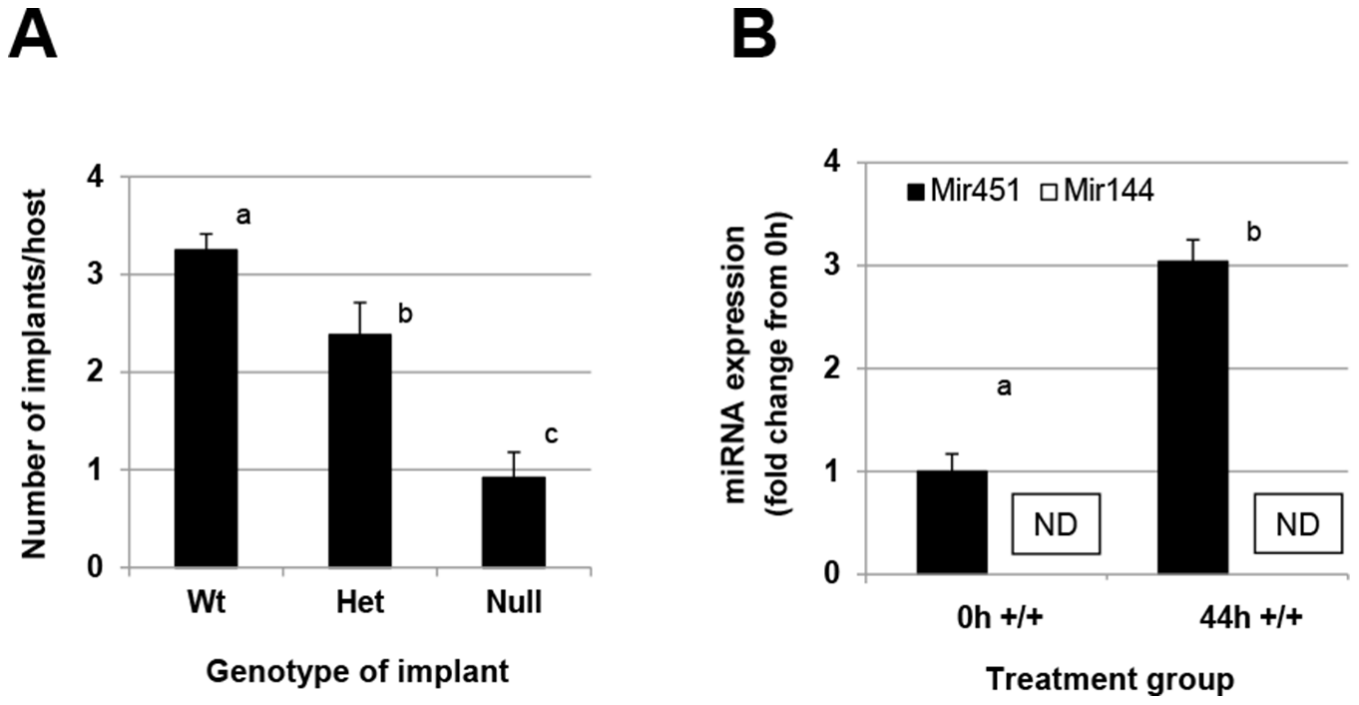
Development of experimental endometriosis is associated with the level of implant *miR-451* expression. Experimental endometriosis was induced and development assessed as described under “*Materials and Methods*”. A. The number of implants of each genotype that developed in wild-type host mice (N = 8/genotype). Data are presented as the mean ± SEM and were analyzed by one-way ANOVA followed by post-hoc analysis. Different letters indicate statistical significance (P<0.05). B. Elevated *miR-451* expression is associated with wild-type “implant” tissue that develops ectopically. Uterine fragments were obtained from wild-type mice at the time (0 h) of PMSG administration or 44 h later (the time of endometrial fragment harvest). *miR-451* and *miR-144* expression was determined by qRT-PCR. Different letters indicate statistical significance (P<0.05) between groups (by unpaired t-test). ND indicates that *miR-144* levels were not detectable by qRT-PCR.

Based upon the initial demonstration that *miR-451* expression was reduced in eutopic endometrium from women with endometriosis, coupled with the postulated factors which *miR-451* may regulate and their role in endometriosis establishment, we hypothesized that the reduced *miR-451* expression by eutopic endometrium may enhance the ability of this displaced endometrial tissue to establish ectopically and develop into endometriosis. To test this hypothesis, we transferred endometrial tissue fragments from *miR-451* ^+/+^, *miR-451* ^+/−^ and *miR-451* ^−/−^ donor mice into 2 to 4 month-old wild-type (*miR-451* ^+/+^) females and assessed the number of implants which developed 4 weeks after transfer. Surprisingly, transfer of tissue fragments from *miR-451* ^−/−^ mice resulted in significantly less implants developing in wild-type recipients compared to the number of developed implants derived from *miR-451* ^+/−^ and *miR-451* ^+/+^ donors (Fig. 2A). qRT-PCR was performed on endometrial fragment tissue from wild-type and *miR-451* ^−/−^ mice to confirm induction of *miR-451* in wild-type mice as well as assess *miR-144* expression. As depicted in Figure 2B, PMSG treatment induced a significant increase in *miR-451* expression in the wild-type mice. *miR-451* expression was not detected in the null mice as expected (data not shown). *miR-144* was not detected in endometrial fragments from wild-type mice regardless of treatment (Fig. 2B), while *miR-144* was not detected in the *miR-451*/*144* ^−/−^ mice as expected (data not shown).

### Endometriotic implant deficiency, not host environment, reduces establishment of disease

**Figure 3:**
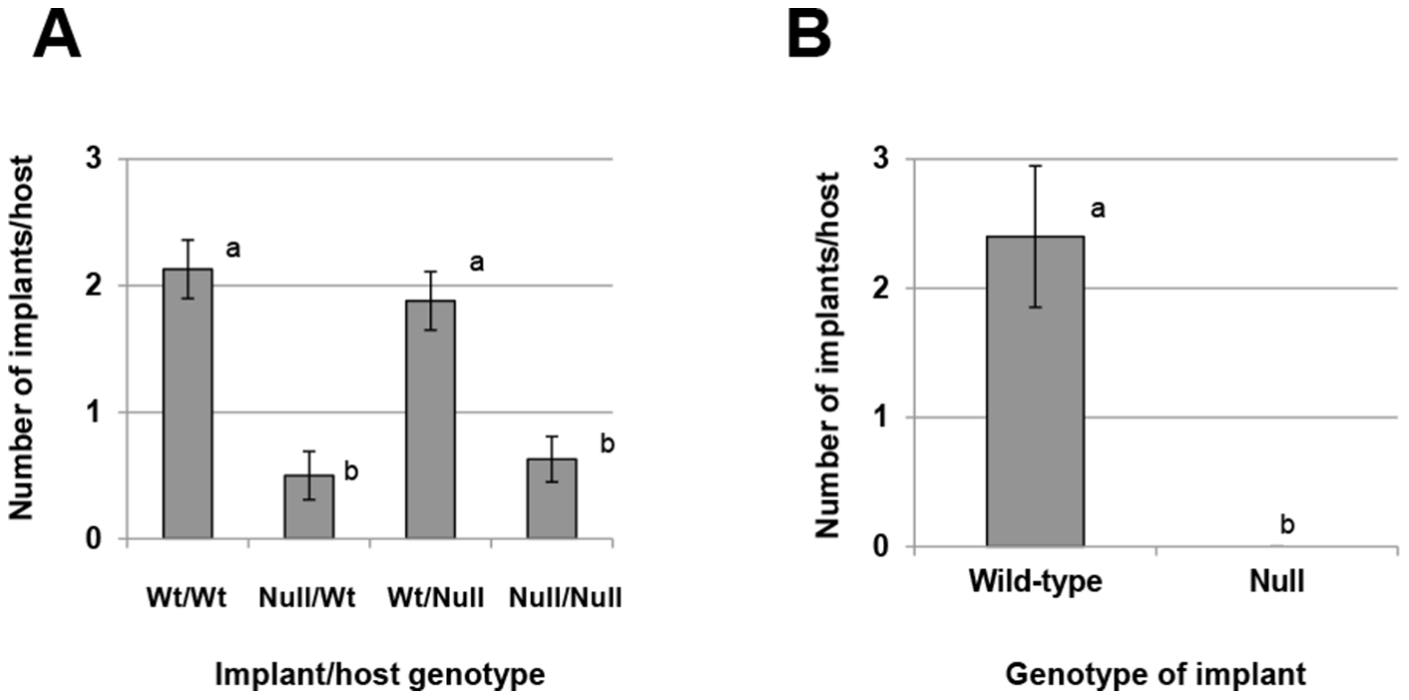
Development of experimental endometriosis is dependent upon implant *miR-451* expression and not host expression. A. Endometriosis was induced in wild-type and *miR-451* null mice using donor tissue from both wild-type and *miR-451* null mice and the number of implants which developed was assessed as described under “*Materials and Methods*.” Data are displayed as the mean ± SEM. Different letters indicate statistically significant differences among the means as determined by one-way ANOVA followed by post-hoc analysis (N = 6 per group). B. *miR-451* wild-type and not *miR-451* deficient implants develop in the same wild-type host. Endometriosis was induced as described under “*Materials and Methods*” in wild-type host mice using both miR-451 wild-type (expressing EGFP) and *miR-451* deficient (non-EGFP) endometrial fragments and implant establishment was assessed. Data are displayed as the mean ± SEM. Different letters indicate statistically significant differences among the means as determined by unpaired t-test (N = 6 per group).

To verify that the reduced ability of *miR-451* deficient tissue to establish ectopically was due to absence of *miR-451* in the implant tissue and to examine if *miR-451* in the host (peritoneum) impacted implant development, we conducted an additional series of studies. In the first series of experiments, endometriosis was induced in wild-type mice which received *miR-451* ^+/+^ or miR-*451* ^−/−^ implant tissue as well as *miR-451* ^−/−^ mice which received *miR-451* ^+/+^ or *miR-451* ^−/−^ implant tissue. Regardless of host genotype, a significantly greater number of *miR-451* ^+/+^ implants developed compared to the number of *miR-451* ^−/−^ implants (Fig. 3A).

In the second series of experiments, we again induced endometriosis using donor tissue from both *miR-451* ^+/+^ (this time wild-type tissue expressed EGFP) and *miR-451* ^−/−^ (non-EGFP expressing) mice and transferred an equal number of fragments of both genotypes into recipient mice. Assessment of implant development and genotype (by both EGFP expression and qRT-PCR of *miR-451*) one week post induction revealed that all mice developed endometriotic implants and that all of the implants were derived from wild-type mice/expressed EGFP (Fig. 3B).

### Impaired ability of *miR-451* deficient uterine tissue to develop ectopically is associated with fibrinogen alpha chain precursor levels

**Figure 4:**
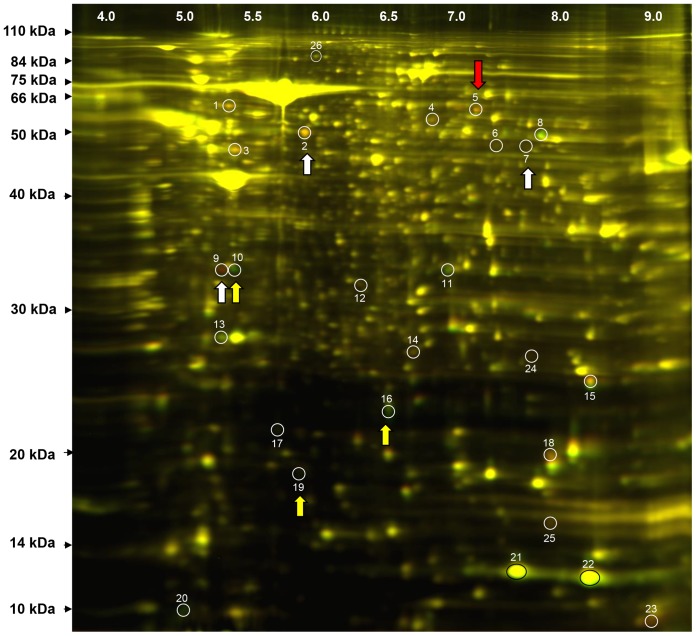
CyDye switch, two dimensional fluorescence difference gel electrophoresis (2D-DIGE) analysis of wild-type and *miR-451* deficient endometrial fragment proteomes. Endometrial fragments were obtained from wild-type (N = 6) and *miR-451* deficient mice (N = 6) as described under “*Materials and Methods*”. Wild-type samples were labeled with Cy3 (green) and *miR-451* deficient samples with Cy5 (red). Samples were then mixed and separated on analytical 2-D DIGE. The resulting gel was scanned and the merged image is shown where red proteins represent proteins whose expression is higher in the *miR-451* deficient tissue and green proteins represent proteins whose expression is higher in the wild-type tissue. Circled and numbered spots represent proteins which were most differentially expressed of which only those indicated by white (up-regulated) or yellow (down-regulated) arrows are reported in Table 1. Red arrow indicates protein #5 which was identified as fibrinogen, alpha polypeptide isoform 2 precursor.

To begin to determine the mechanisms by which *miR-451* deficiency may impair the establishment of ectopic endometrial tissue fragments, we isolated endometrial fragments from both *miR-451* ^+/+^ and *miR-451* ^−/−^ donors (N = 6/genotype, prior to transfer into recipients) and assessed protein profiles using two-dimensional differential in-gel electrophoresis (DiGE). From the hundreds of proteins expressed, twenty-five were further analyzed by MALDI-TOF (MS) and TOF/TOF (tandem MS/MS; Fig. 4 and Table S1). Of these 25 proteins, we focused on the seven most significantly differentially expressed proteins (>1.5 fold change up or down) between *miR-451*−/− and wild-type tissues. Included in the inclusion criteria for these proteins were purity (some protein spots may contain two proteins) and identification as a single protein (some spots were identified with C.I.% of 100% but could not be identified as a single protein). After identification of these proteins and their reported biological functions, we focused on fibrinogen alpha polypeptide isoform 2 precursor (spot #5) as this protein may potentially modulate cell adhesion based on its RGD amino acid content [26] as well as neoangiogenesis [27]; both cellular events which contribute to ectopic implant survival and establishment.

**Table 1:** Most significantly modulated proteins in uterine fragments from *miR-451* deficient mice compared to wild-type counterparts.

Spot Number	Protein ID	Accession number	MW	Pir/Pid^a^	Fold change^b^
2	Keratin, type II cytoskeletal 7	K2C7	50,678	5.67/5.67	1.52
5	Fibrinogen, alpha polypeptide	giI33563252	61,288	7.16/7.16	1.85
	isoform 2 precursor				
7	NADH dehydrogenase [ubiquinone]	NDUV1	50,802	8.51/7.74	1.94
	flavoprotein 1 (mitochondrial)				
9	Glyoxylase domain-containing protein 4	GLOD4	33,296	5.28/5.19	5.34
10	Glyoxylase domain-containing protein 4	GLOD4	33,296	5.28/5.28	−2.84
16	Transgelin	TAGL	22,561	8.85/6.46	−2.13
19	Bis (5′-nucleosyl)-tetraphosphatase	AP4A	16,979	5.87/5.87	−1.93

a  =  Pir/Pid  =  reported isoelectric point/detected isoelectric point. Differences between the reported and detected isoelectric points may be due to alterations in post-translational modifications such as Phosphorylation which decreases the PI.

b  =  Fold change is expressed as level of each protein detected in the *miR-451* null/*miR-451* wild-type samples.

### *miR-451* endometrial deficiency is associated with altered fibrinogen alpha chain expression and elevated plasmin activity

**Figure 5:**
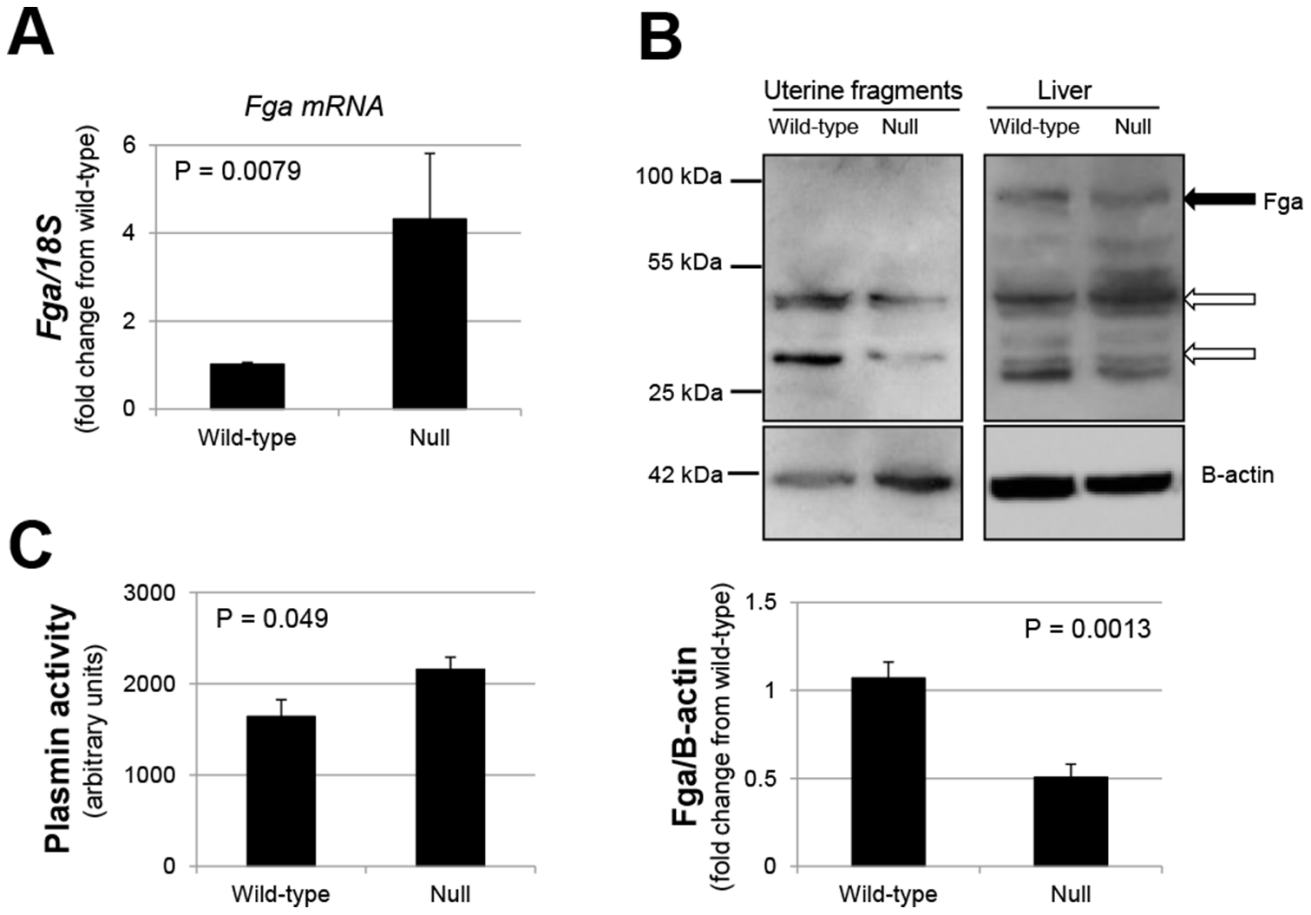
Fibrinogen transcript, protein and active plasmin expression in endometrial fragments of wild-type and *miR-451* deficient mice. Fibrinogen alpha chain (Fga) transcript (A) and protein (B) were analyzed by qRT-PCR and Western blot expression, respectively. Data are representative of 5 observations per endpoint per genotype (N = 5). *Fga* mRNA levels were not normally distributed and were analyzed using Mann-Whitney tests. Bar graph data are displayed as the mean ± SEM. In B), black arrow indicates molecular weight (95 kDa) of fibrinogen alpha chain while white arrows indicate major Fga fragments of approximately 42 kDa and 34 kDa. C) Plasmin activity was determined in uterine fragments from wild-type and *miR-451* null mice as described under “*Materials and Methods*.” Data are displaed as the mean ± SEM and are representative of 6 separate observations per genotype (N = 6). P values are indicated in each figure and data were analyzed by unpaired t-tests in B and C. As Fga mRNA data (A) were not normally distributed, data was analyzed using the non-parametric t-test (Mann-Whitney).

As fibrinogen alpha polypeptide isoform 2 precursor is liberated from the fibrinogen alpha chain (Fga), we first examined if *Fga* transcript expression differed between genotypes. As depicted in Figure 5A, *miR-451* deficient endometrial fragments expressed significantly higher levels of *Fga* mRNA compared to wild-type counterparts. To determine if this increase in transcript was associated with increased Fga protein expression, Western analysis was performed. In contrast to transcript expression, total Fga protein levels (quantitation of all bands as a sum) were significantly lower in endometrial fragments from *miR-451* deficient mice (Fig. 5B). Two major bands were detected with approximate molecular weights of 34- and 42 kDa, but no band consistent with the parent Fga protein (approximately 95 kDa) or fibrinogen alpha polypeptide isoform 2 precursor were detected (Fig. 5B). In contrast, the 95 kDa Fga as well as multiple bands including the 34- and 42-kDa protein detected in endometrial fragments were detected in mouse liver (the major source of fibrinogen) from both wild-type and null mice.

As the 34- and/or 42-kDa bands detected by the Fga antibody may represent cleavage products of the 95 kDa protein, we assessed plasmin activity in endometrial fragments from mice of both genotypes. Endometrial plasmin activity was significantly greater in the endometrial fragments from *miR-451* deficient mice compared to fragments from wild-type mice and was associated with reduced Fga protein levels (Fig. 5C).

### RGD cyclic peptide impairs human endometrial stromal cell spreading *in vitro* and reduces endometriosis establishment *in vivo*

**Figure 6:**
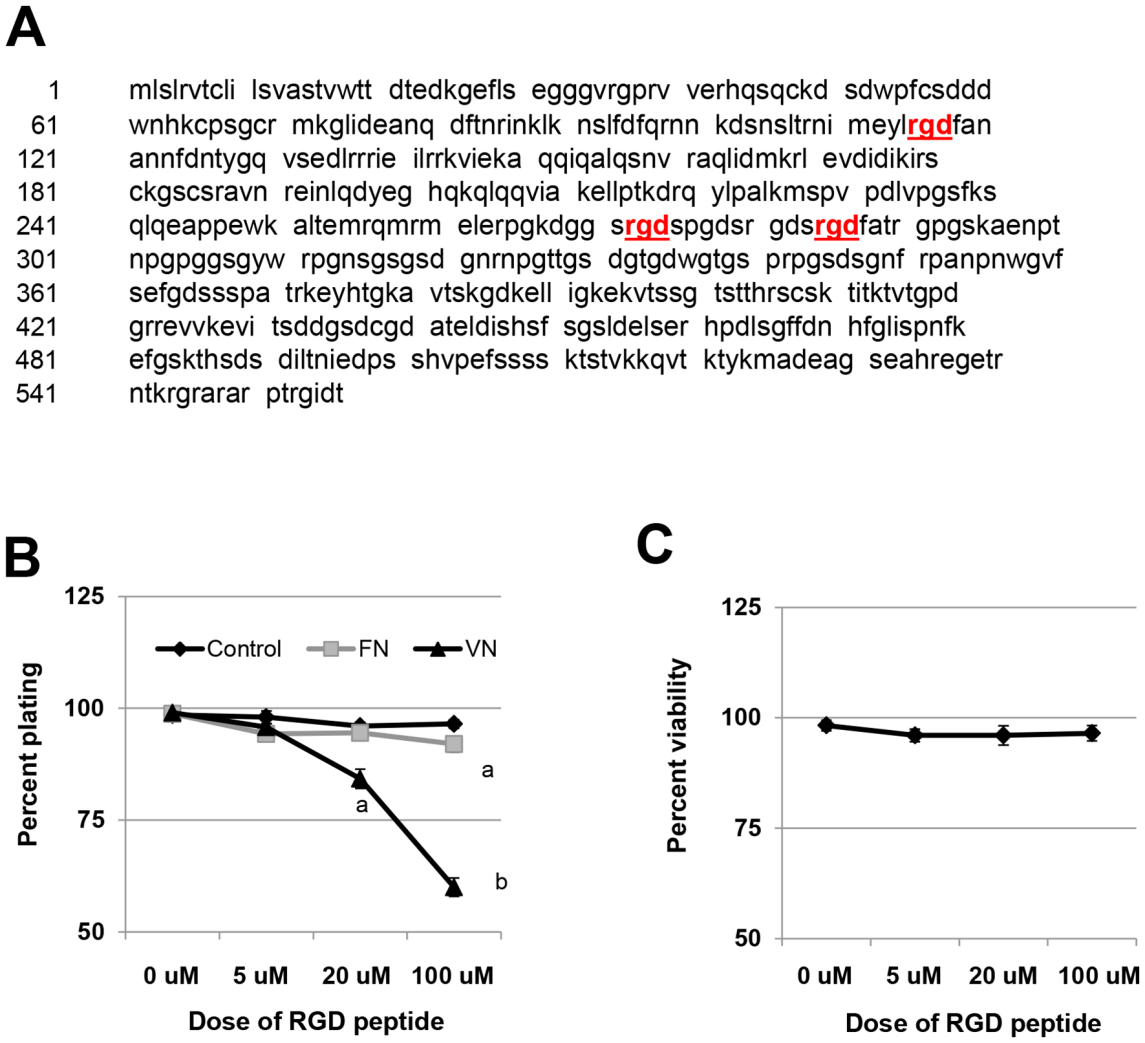
Fibrinogen alpha polypeptide isoform 2 precursor contains multiple cell adhesion motifs which modulate cell adhesion in vitro. A. Protein sequence of mouse fibrinogen alpha polypeptide isoform 2 precursor. Three RGD sequences were detected in the protein sequence and are indicated in red underlined text. B. Pre-treatment of the immortalized human endometrial stromal cell line, t-HESC with cyclic RGD peptide inhibits in vitro cell spreading and survival. T-HESC cells were treated and cell adhesion, spreading and survival assessed as described under “*Materials and Methods*.” Data are displayed as the mean ± SEM. Different letters indicate statistically significant differences among the means within each substrate as determined by one-way ANOVA followed by post-hoc analysis (N = 4 separate experiments). C. Pre-treatment with RGD cyclic peptide does not induce cell death. Data are displayed as the mean ± SEM and are representative of 4 separate experiments (N = 4 separate experiments). Means are not significantly different among the different doses of RGD peptide.

We postulated that the reduced ability of *miR-451* deficient tissue to establish ectopically may be due to elevated expression of fibrinogen alpha chain precursor as this protein contains three RGD cell adhesion motifs (Fig. 6A) which in theory would impede cell to cell adhesion and/or viability necessary for ectopic implant establishment and survival. To begin to assess this possibility, we first elected to assess endometrial stromal cell adhesion/spreading *in vitro* to determine if the RGD motif could prevent the stromal cells from binding with vitronectin and/or fibronectin; extracellular matrix proteins which also contains the RGD sequence and are postulated to promote cell to cell adhesion via integrin αvβ3 [28] and/or α1β5; integrins, which are expressed on endometrial tissue/cells [28], [29]. Pre-incubation of stromal cells with RGD peptide for 1 h resulted in a significant, dose-dependent decrease in stromal cell attachment and spreading on vitronectin-coated tissue culture plates as well as on fibronectin-coated tissue culture plates (to a much lesser degree compared to vitronectin) but not to bovine serum albumin-coated tissue culture plates (Fig. 6B). This reduction in spreading was not associated with an initial decrease in cell viability after the pre-incubation with RGD peptide (Fig. 6C).

**Figure 7:**
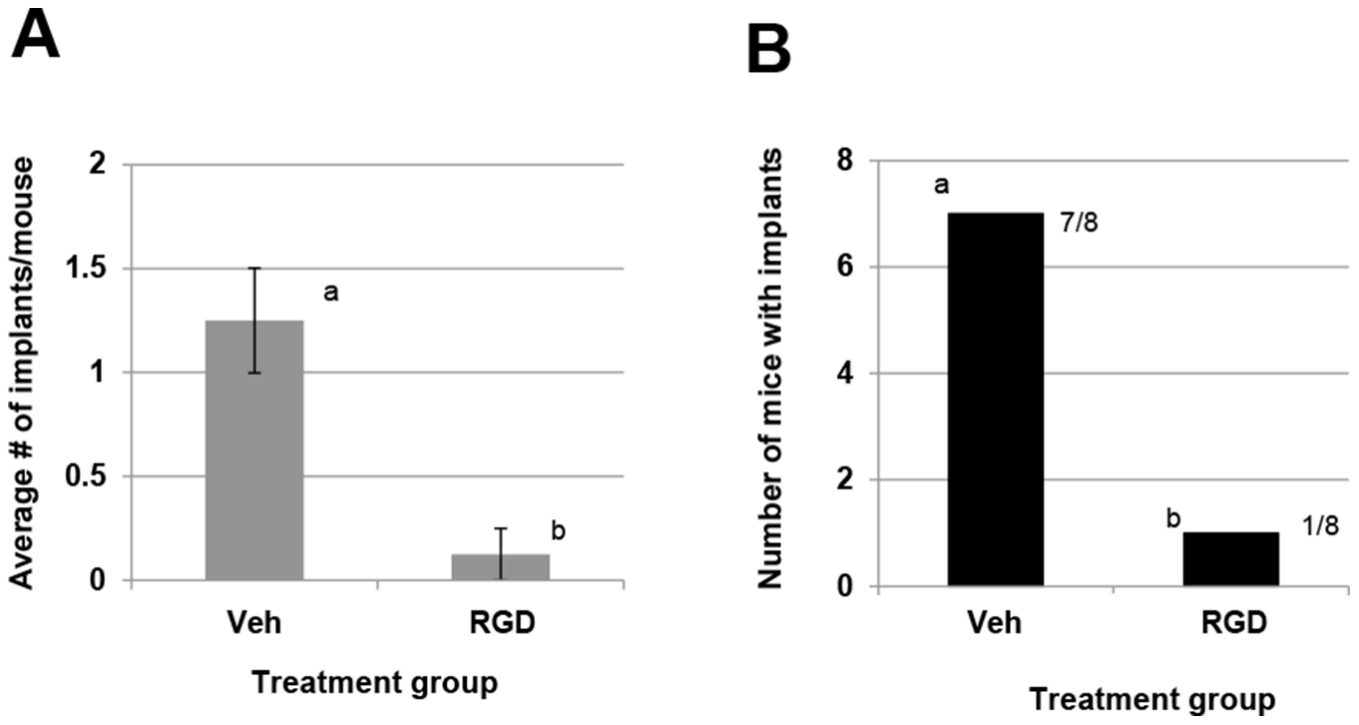
RGD cycle peptide inhibits development of experimental endometriosis. Experimental endometriosis was induced in wild-type mice with wild-type endometrial fragments which were pre-treated with either vehicle (Veh) or RGD peptide (RGD) and development of ectopic lesions was assessed as described under “*Materials and Methods*.” Data are displayed as the mean ± SEM. Different letters indicate statistically significant differences among the means between treatments as determined by unpaired t-tests (N = 8/group). B Chi square analysis of the proportion of mice which received implant tissue pre-treated with either vehicle (Veh) or RGD cyclic peptide (RGD).

To determine if the RGD cyclic peptide could also influence the ability of endometrial fragments to establish ectopically *in vivo*, we pretreated endometrial fragments from wild-type mice with 100 µM of cyclic RGD peptide or vehicle for 1 h then transferred the fragments to wild-type recipients (N = 8 per treatment). Two weeks post endometriosis induction, the number of implants which developed were assessed (Fig. 7). Mice receiving endometrial fragments pre-treated with the RGD cyclic peptide had significantly fewer implants compared to mice receiving vehicle pre-treated implants (Fig. 7A). Further, significantly less mice which received the RGD peptide pre-treated implants developed implants (1/8 vs. 7/8 by chi square analysis; Fig. 7B).

## Discussion

The mechanisms by which endometriosis develops are poorly understood, but retrograde menstruation is proposed to play a role in the development of the disease. However, as almost all women exhibit some degree of reverse menses [3], [4], there is strong belief that other contributing factors must exist. Considerable attention has focused on the alterations in eutopic endometrium from women with endometriosis which may make this tissue (and therefore these women) more likely to develop ectopically upon seeding into the peritoneal cavity during reverse menstruation. Based upon an initial report by Pan and colleagues [9] which suggested that *miR-451* expression in eutopic endometrium is significantly reduced compared to eutopic endometrium from women free of endometriosis, coupled with the belief that *miR-451* regulates many factors which may play a role in the initial establishment of retrogradely shed endometrial fragments into endometriosis, we set out to determine if the level of eutopic endometrial (uterine) tissue *miR-451* expression influenced the ability of this tissue to establish ectopically.

To do so, we utilized mice deficient for *miR-451/144* expression to examine the function of this miRNA(s) in the initial establishment of endometriotic implants. Loss of *miR-451/144* expression was associated with a reduced ability of the tissue to establish ectopically. These observations may support the notion that reduced *miR-451* expression in eutopic endometrium from women with endometriosis is a result of the disease, not a cause for its establishment as reduced expression within this eutopic tissue did not make it more apt to develop ectopically. In addition to the current study, this postulate is also supported by the observation that induction of endometriosis in baboons is associated with a reduction of eutopic endometrium *miR-451* expression after the disease develops (unpublished data; Fazleabas and colleagues). Taken together, it appears that the reduced levels of *miR-451* in eutopic endometrium of women with endometriosis do not functionally enhance the ability of this tissue to establish ectopically and that the reduced levels of *miR-451* detected in eutopic endometrium from women (and baboons) with endometriosis, may be a result of already established ectopic implants/presence of existing endometriosis.

To begin to assess the mechanisms by which *miR-451* deficiency influences the ability of endometrial fragments to establish ectopically, we performed 2D-DiGE. From the differentially expressed proteins identified, we further focused on fibrinogen alpha polypeptide isoform 2 precursor which is an approximate 62 kDa protein derived from processing of the fibrinogen alpha chain (Fga). Fibrinogen alpha polypeptide isoform 2 precursor contains three RGD sequences (compared to the RGD content of vitronectin (VN; 1 RGD sequence). Based upon RGD content, fibrinogen alpha polypeptide isoform 2 precursor, would be more likely to displace *in vivo* VN binding to its respective integrin, αvβ3 allowing for adhesion/cell attachment. Our *in vitro* and *in vivo* studies using the RGD peptide antagonist, cyloRGDfV confirm that adhesion of endometrial stromal cells and mouse endometrial implant tissue occur via an RGD-dependent mechanism and that VN may be a major component of this adhesive mechanism.

αVβ3 is over-expressed in endometrial tissue from women with endometriosis and is proposed to play a role in the development of the disease [30]. However, there is little evidence on the actual role of this integrin in the cell-cell mechanisms for establishment of ectopic lesions *in vivo*. A single report demonstrated that administration of an αVβ3 blocking antibody inhibits growth of endometriosis in a mouse model for the disease [31] but also reported an increase in lesion weight. As synthetic peptides containing the RGD motif are known to induce apoptosis [32], these agents may represent a novel approach to targeting existing disease and/or preventing the establishment of new ectopic lesions.

To begin to explore the mechanisms for the elevated expression of fibrinogen alpha polypeptide isoform 2 precursor we started with assessment of *Fga* mRNA expression, which this polypeptide is derived from. *Fga* transcript expression in endometrial fragments from *miR-451/144* null mice was elevated compared to wild-type counterparts. This elevation is not due to loss of *miR-451* as the 3′UTR of *Fga* does not contain a binding site for *miR-451*. In contrast, *miR-144* is proposed to target *Fga* based upon miRNA target prediction resources. However, the increase expression of *Fga* transcript in the *miR-451/144* null tissue compared to the wild-type tissue cannot be attributed to loss of *miR-144* expression in the null tissue as both null and wild-type endometrial fragments did not express detectable *miR-144*. It may be plausible that the elevated levels of *Fga* transcript expression in the *miR-45/144* null tissue may be due to loss of expression of the repressive miRNA which results in an up-regulation of target transcript.

In addition to transcript expression, we also examined Fga protein expression in endometrial fragments from *miR-451* null and wild-type mice. Fga was assessed as opposed to fibrinogen alpha polypeptide isoform 2 precursor as there currently is no antibody available for this protein fragment. We found that in contrast to *Fga* mRNA expression, total Fga protein levels (quantitated as all bands recognized by the Fga antibody) were significantly lower in the *miR-451* null tissue. Further, unlike in liver samples, endometrial samples did not express the full length Fga protein (95 kDa) but did express two major fragments at approximately 34- and 42 kDa. The absence of the 95 kDa Fga protein in uterine tissue may have been due to cleavage/degradation of the parent chain. This postulate prompted us to examine levels of plasmin activity in these tissues. Fibrinogen cleavage by plasmin releases several fragments from the fibrinogen molecule [33]. Previously, we demonstrated that estrogen primed uterine tissue expressed elevated plasmin activity [24] and a similar result was obtained in the current study in PMSG-primed mice (which will have endogenously stimulated estrogen-primed uterine tissue). Thus, elevated plasmin activity in the endometrial fragments of *miR-451* null mice may play a role in the degradation/reduction of total Fga protein characteristic of these fragments leading to an overall decrease in the expression of total Fga fragments in the null endometrial tissue. We propose that in both wild-type and null endometrial tissue, elevated plasmin activity may lead to loss/decrease in the relative abundance of the 95 kDa Fga molecule, while in the null tissue itself the significantly higher plasmin activity may lead to further selective degradation of the 34- and 42 kDa fragments (as seen in Fig. 5) but increased expression in the fibrinogen alpha polypeptide isoform 2 precursor (as presented in Fig. 4).

As this is the first study to assess the functional role of a miRNA in endometriosis using a genetically modified mouse model, we feel that there are several points which should be discussed in the context of this model. First, the observation in the current study that reduced *miR-451* expression is associated with a decrease in the establishment of ectopic endometriotic tissue may be due to indirect, down-stream effects of *miR-451/144* deficiency. In the current model, mice are primed with pregnant mare serum gonadotropin (PMSG, an FSH like analog) to induce endogenous estrogen production and then sacrificed approximately 44 h later. While we previously demonstrated that estradiol induces a significant increase in mouse uterine *miR-451* expression as early as 2 h post-administration [23], in the PMSG-primed model, systemic estradiol levels increase as early as 12 h post PMSG administration and remain elevated until around the time of ovulation (about 52–56 h post PMSG administration; unpublished data). Thus, it is possible that any alterations in the genome which result from the loss of *miR-451* had occurred early after PMSG administration and that by 44 h post administration, the protein profiles we assessed were a result of the downstream or indirect effects of *miR-451* deficiency. This may explain why, of the seven most significantly up-regulated proteins in the *miR-451* ^−/−^ uterine fragments, none are putative direct targets of *miR-451* post-transcriptional regulation based upon bioinformatics programs such as TargetScan and Miranda which predict miRNA targets.

A second point of discussion relates to the biogenesis of *miR-451* and *miR-144* mature forms. *miR-451* is co-expressed with *miR-144* on a bicistronic precursor RNA (pre-miR) that is particularly abundant in red blood cell precursors, but also found at lower levels in normal tissues and some cancers [34]–[37]. Thus, it was surprising that in PMSG-primed uterine tissue fragments that *miR-451* was expressed but *miR-144* was undetectable (based upon the limitations of our experimental design). Differences in *miR-144* and *miR-451* despite their co-expression on a single pre-miR may be due to differences their subsequent processing. Specifically, *miR-451* primary microRNA (pri-miRNA) is uniquely processed by Argonaut 2, independent of Dicer [38], [39]. Thus, this observation may suggest additional differential processing of the pre-miRNAs or novel transcriptional regulation resulting in enrichment of *miR-451* in murine uterine tissue.

The original objective of our study coupled with the observation that *miR-451* deficient tissue was less apt to establish ectopically led us to concentrate on potential cell adhesion/implant adhesion mechanisms. However, it was interesting to note that the most-significantly upregulated proteins in *miR-451* deficient endometrial fragments were those of mitochondrial origin/function (Nduv1 and Glod4). Mitochondrial DNA mutations and alterations associated with endometriosis were first reported in 2005 by Kao and colleagues [40]. Subsequent studies have proposed that endometriotic eutopic endometrial mitochondrial biomarkers may be used for diagnosis [41]–[43] and that mitochondrial DNA polymorphisms may be associated with susceptibility to the disease [44]. More recently, alterations in the mitochondrial displacement loop (D-loop) [45] and in the mitochondrial membrane complex I (MMC-I also known as NADH:ubiquinone oxidoreductase) [46] have been proposed to be inheritable risk factors for endometriosis as well. To the best of our knowledge, the potential role of *miR-451* or any other miRNA in regulating Nduv1 and/or Glod4 expression has not been described and deserves further exploration.

In summary, disruption of *miR-451* expression in endometrial tissue impairs the ability of this tissue to establish ectopically in a mouse model of endometriosis. Loss of *miR-451* expression was associated with differential expression of proteins. Of these proteins, fibrinogen alpha polypeptide isoform 2 precursor, which contains multiple RGD/cell adhesion peptide sequences, was significantly elevated in *miR-451* deficient endometrial tissue. Elevated levels of this Fga polypeptide are associated with altered expression of the parent Fga transcript and protein in the *miR-451* deficient tissue. Pre-treatment of an immortalized human endometrial stromal cell line as well as donor endometrial fragments with a cyclic RGD peptide, respectively, inhibits *in vitro* cell spreading and survival and establishment of endometriotic implants *in vivo*. Collectively, these observations may be interpreted to suggest that the reduced expression of *miR-451* characteristic of eutopic endometrium from women with endometriosis does not enhance the ability of retrogradely displaced endometrial tissue to develop ectopically and that the reduced levels of *miR-451* expression in eutopic/retrogradely shed endometrium may be a result of, not a causative factor, in the development of the disease. Lastly, as administration of cyclic RGD peptide reduced ectopic endometrial lesion establishment, the use of these and/or similar RGD peptides may prove useful in the prevention of recurrent endometriosis development.

## Supporting Information

Table S1 **Raw data on protein peptide summary and protein identification from wild-type and** ***miR-451*** **null endometrial fragments post PMGS administration.**
(XLS)
